# Assessing health-care providers’ readiness for reporting quality and patient safety indicators at primary health-care centres in Lebanon: a national cross-sectional survey

**DOI:** 10.1186/s12960-015-0031-5

**Published:** 2015-05-22

**Authors:** Mohamad Alameddine, Shadi Saleh, Nabil Natafgi

**Affiliations:** Department of Health Management and Policy, Faculty of Health Sciences, American University of Beirut, PO Box 11-0236, Riad El-Solh, Beirut 1107 2020 Lebanon; Department of Health Management and Policy, College of Public Health, University of Iowa, CPHB - N277, 145 N. Riverside Dr., Iowa City, IA 52242 USA

## Abstract

**Background:**

Successful endorsement of quality indicators hinges on the readiness and acceptability of care providers for those measures. This paper aims to assess the readiness of care providers in the primary health-care sector in Lebanon for the implementation of quality and patient safety indicators.

**Methods:**

A cross-sectional survey methodology was utilized to gather information from 943 clinical care providers working at 123 primary health-care centres in Lebanon. The questionnaire included two sections: the *first* assessed four readiness dimensions (appropriateness, management support, efficacy, and personal valence) of clinical providers to use quality and safety indicators using the Readiness for Organization Change (ROC) scale, and the *second* section assessed the safety attitude at the primary care centre utilizing the Agency of Health Research and Quality (AHRQ) Safety Attitude Questionnaire (SAQ)-Ambulatory version.

**Results:**

Although two thirds (66 %) of respondents indicated readiness for implementation of quality and patient safety indicators in their centres, there appear to be differences by professional group. Physicians displayed the lowest scores on all readiness dimensions except for personal valence which was the lowest among nurses (60 %). In contrast, allied health professionals displayed the highest scores across all readiness dimensions. Generally, respondents reflected a positive safety attitude climate in the centres. Yet, there remain a few areas of concern related to punitive culture (only 12.8 % agree that staff should not be punished for reported errors/incidents), continuity of care (41.1 % believe in the negative consequences of lack in continuity of care process), and resources (48.1 % believe that the medical equipment they have are adequate). Providers with the highest SAQ score had 2.7, 1.7, 7 and 2.4 times the odds to report a higher readiness on the appropriateness, efficacy, management and personal valence ROC subscales, respectively (*P* value <0.01). Nurses displayed relatively lower odds of readiness across all other ROC subscales as compared to all other providers.

**Conclusion:**

Health-care providers at the primary health care (PHC) centres in Lebanon are ready to engage in employing quality and patient safety indicators. This is a key finding given the active efforts by the MoPH to strengthen the quality culture in the PHC sector through various strategies.

## Introduction

Primary health-care centres (PHCCs) constitute the setting in which local communities are most likely to receive ‘practical, scientifically sound and socially acceptable’ care that is both sustainable and universally accessible [1]. Since the late seventies, primary health care (PHC) has been envisioned, with the Alma Ata Declaration, as an integral part of the health system and a cornerstone in the social and economic growth of communities [1]. Thirty years after the Declaration that officially highlighted the significance of PHC in addressing the health-care needs of populations in 1978, PHC was again at the centre stage of the international health-care agenda with the publication of the 2008 World Health Report ‘Primary Health Care: now more than ever’ [2].

This renewed interest in PHC has been coupled with an increased attention to patient safety and quality of care. The eminent Institute of Medicine (IOM) report ‘To Err is Human’ was the tipping point highlighting quality and patient safety as a priority for health-care organizations [3]. Although the report mainly examined the issue in the United States hospital sector, the concerns highlighted and the subsequent recommendations extend beyond the boundaries of hospitals and are indeed applicable to other countries around the globe, including low- and middle-income countries where different strategies have been employed in an attempt to enhance quality and achieve better health-care outcomes [4].

One of the strategies is the enactment of a comprehensive multi-track quality-enhancing strategy that includes improvement of performance through the development and implementation of quality and patient safety indicators to identify areas in which improvement is required [4]. This would not only help identify potential areas of improvement but also support priority setting efforts with subsequent mobilization of resources towards those priorities [4, 5]. Moreover, employing quality indicators contribute to enhanced quality of work, improved regulation and the establishment of a steadfast system of accountability [6]. However, it is important when devising and establishing indicators to understand and identify the various stakeholders involved, including first and foremost health-care providers [7]. Therefore, the assessment of their readiness for the implementation of quality and safety indicators is critical prior to the development and implementation of any quality assessment initiative.

### Theoretical framework

Imperative organizational change cannot be realized without the consideration of the willingness, qualification and readiness of the organization’s human resources, who are the major factor that either promote or hinder such change [8–10]. The importance of determining the willingness and capacity to adopt organizational and work-process modifications in order to ameliorate the resistance faced during implementation of an organizational change process is well established [11].

**Fig. 1:**
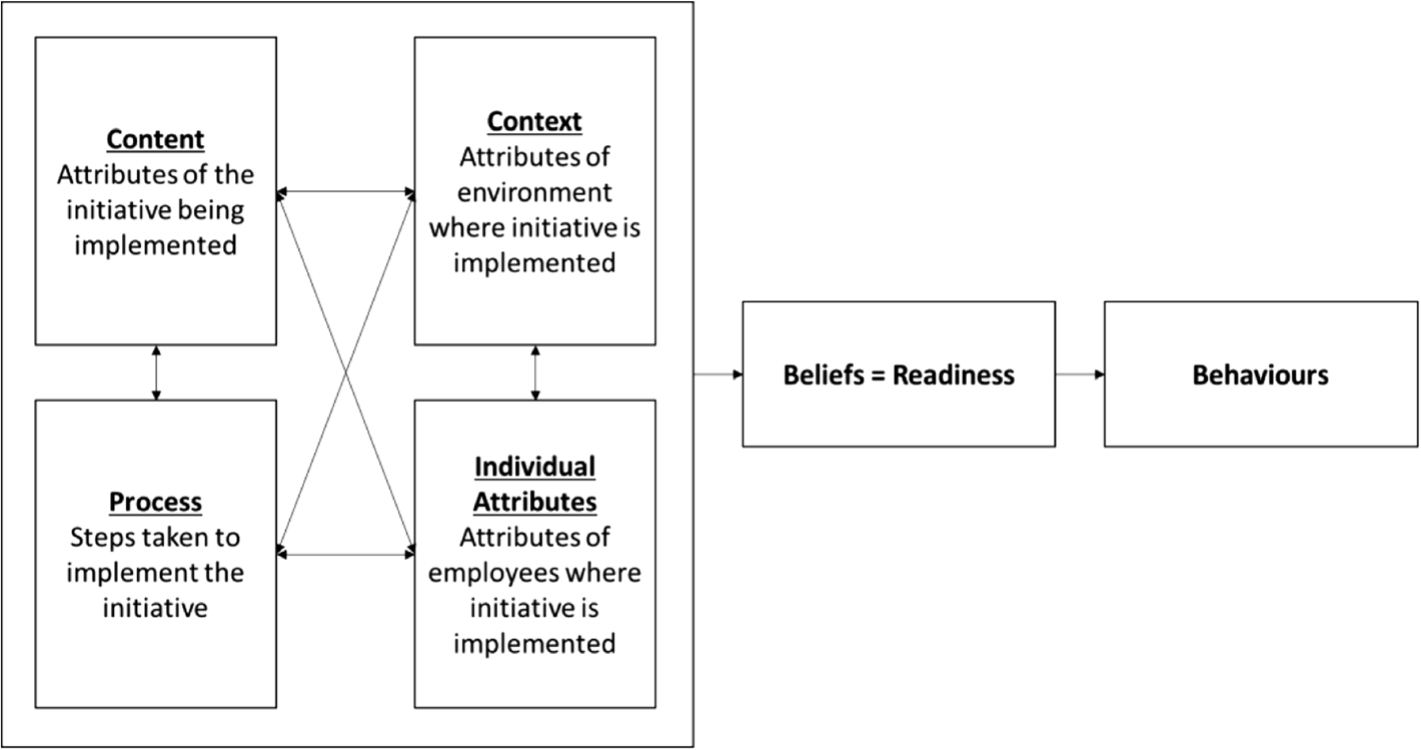
Conceptual framework of the relationship between content, context, process and individual attributes with readiness to change. Source: Holt et al. [14]

Several instruments have been developed to assess readiness prior to the introduction of organizational change in different settings [8, 12–16]. One of the most prominent and widely used measures is that developed by Holt et al. [14]. Holt and colleagues base their Readiness for Organization Change (ROC) scale on four dimensions that are critical for any successful change, namely the following: change content, change context, change process and individual attributes (Fig. 1) [14]. The change content refers to the particular attributes of the change being implemented (introduction of quality and patient safety measures). This may include the number and type of measures (structural, process, outcome) or domain of measures (diabetes management, hypertension, safety, etc.). The change context describes the characteristics of the PHCC or the environment where the change is being introduced, namely administrative, technical and structural conditions. The third perspective is process, which reflects the steps followed during the implementation phase of the introduced change. The last perspective is the individual attributes of the providers that will be responsible for executing the change. This comprehensive model provides a conceptual framework that shapes the ROC scale utilized in this paper as it assesses characteristics that influence readiness and build the foundation for adoptive behaviours.

With that, any change, minor or major, needs to assess the readiness of an organization’s human resources to change, and in case such readiness is absent, serious preparations are vital to develop and inculcate readiness within staff; otherwise, the organization will face major losses in resources, such as time and money, and fail to implement the desired change [9]. Due to this, initial investments to assess and promote readiness for change may prove to have major savings later on during implementation, simply due to the averted resistance of employees [9].

Various factors exist that influence the ability of human resources for health to accept readiness for change. Empowering employees, their level of confidence in their work colleagues and the degree to which employees believe that the organization is capable of achieving the set out changes [11] are established factors that determine the extent of success of change implementation. Other factors that are deemed as facilitating factors to change are having organizational policies that easily accommodate for employee needs [17], in addition to clarifying the awards of adhering to the change [18]. Having said that, readiness can be achieved by inculcating the urgent need for organizational change within employees, by assimilating employees into the change process by being transparent and fully communicating the intentions and process of change and by establishing a benchmark or level of performance needed to be achieved in order to realize the needed/desired change [9, 13]. Preparing employees for change and enhancing their readiness may also require investing in increasing their skills and knowledge [9] to facilitate their task of adhering to new guidelines, procedures and levels of performance. The reason behind undertaking strong initial investments in change readiness assessment and promotion is that if readiness does not exist the chances of failure of adhering to new modes of performance are significant [9]. Hence, it is very important to understand all obstacles and weaknesses to quality indicator implementation prior to their enforcement within PHCs [13], and a readiness assessment may be the tool for this.

### Context

Over the past strenuous decades, the Lebanese health-care system has endured through times of extensive political instability and volatile security situations. The lack of coordination among various stakeholders rendered the health-care system suffering from intermittent, and in many cases insufficient, supply of financial and material resources needed to fulfil its fundamental goal of providing needed health services and improving the health status of the Lebanese population. In 1996, a national primary health-care network (the Network) of 29 health centres was established, of which 19 were NGO-owned, 8 were affiliated with the Ministry of Public Health (MoPH) and 2 with the Ministry of Social Affairs (MoSA) [19, 20]. The Lebanese Ministries of Public Health (MoPH), Interior and Municipalities (MoIM) and Social Affairs (MoSA) joined efforts in refurbishing Lebanon’s health infrastructure and network by means of the endeavours taken to expand and increase the capacity of the network [20]. This network grew steadily over the years through contractual agreements with local non-governmental organizations (NGOs), local governmental entities and religiously affiliated agencies to 165 centres in 2012 [21, 22]. The national primary health-care network also offered employment to a large number of health-care providers on a full-time, part-time, casual and voluntary basis. Employment of health-care providers varied by professional group; while the vast majority of nurses and allied health professionals are salaried professionals working on a full-time basis in these centres, the majority of physicians are working on part-time, casual or voluntary bases.

These centres contributed to enhancing people’s access to needed health services due to their vast geographic distribution and relieve the heavy financial commitment by Lebanon to meet its health-care needs. Presently, the Lebanese health-care system, via the MoPH, is venturing upon various initiatives to build the quality, capacity and breadth of the health services provided by the national network. Also, the ministry has recently initiated an accreditation programme for PHCCs in Lebanon. The first steps of the process were taken in 2010 by carrying an external audit through an international accrediting agency of three centres in the network [23]. The second phase of the primary care accreditation scheme was launched in April 2011 where audits were expanded to include 23 additional PHCCs [22, 24]. These audits were accompanied and followed by a series of capacity-building workshops with PHCC managers and providers to coach them on the importance of accreditation and performance improvement activities. Yet, the thinking so far has been majorly centred on accreditation with expressed interest to expand to other complementary quality-enhancing initiatives. In that context, understanding providers’ preference is key since the potential adoption of quality and patient safety indicators will require changes to several centre activities. These include strengthening of the health information systems from a data-capturing standpoint (e.g. forms and information technology infrastructure). In addition, the clinical-data-recording practice among health-care providers has to be solidified and validated, through capacity building, sharing of information on indicators, etc. Finally, a culture that is open to performance improvement has to be gradually instilled. All of these are considerable changes to the current context that providers will have to consider in assessing their readiness.

### Study objectives

The objective of this study is to assess the readiness of clinical providers within PHCCs to adopt quality indicators. This study also aims to reveal the safety climate and overall capacities of PHCCs and their employees to fulfil other health-care quality improvement requirements, such as those required for the national accreditation of primary health-care organizations in Lebanon. This assessment is original because it exposes areas that require new and alternative policies in the Lebanese primary health-care system in order to promote and facilitate the aspiration to optimal patient safety.

## Methods

### Study design, setting and population

A cross-sectional survey methodology was utilized to gather information from clinical care providers working at PHCCs in Lebanon. The study population comprised a total of around 2 140 health-care providers practising in the 123 centres (out of a total of 165) that are part of the Ministry of Public Health (MoPH) primary care network and are fully compliant with MoPH regulatory requirements. Forty-two centres were classified by the Ministry as non-compliant to the PHCC requirements of the MoPH and were under probation. Since those centres did not satisfy the requirements of the MoPH criteria for PHCCs, they were excluded from the survey as they would probably not be included in the implementation of the change (introduction of quality and patient safety indicators). Centres belonging to the national network are subject to a contractual agreement with the MoPH and benefit from considerable support in terms of guidelines and health education materials, training activities, vaccines, drugs, medical supplies and equipment. The centres provide a comprehensive package of health services, including immunization, essential drugs, cardiology, paediatrics, and reproductive health along with other health promotion activities. Each health centre has a defined catchment area with an average of 30 000 inhabitants, with few centres covering around half that population in rural areas [20]. All clinical care providers including physicians, dentists, nurses, technicians, nutritionists, pharmacists, social workers and midwives working at least one shift per week were eligible to participate in this study and therefore were asked to complete the survey. Directors, secretaries, clerical assistants and other administrative and support staff were excluded.

Sample size estimates revealed that for a confidence level of 99 %, and a response distribution of 50 %, the desired sample size should be 699 providers to give results accuracy with a margin of error of 4 %. The study approached all providers with the aim to reach a minimum response rate of 33 % (corresponding to 707 providers needed for the aimed statistical significance). The study was approved by the American University of Beirut Institutional Review Board.

### Study protocol

Survey questionnaires along with an information sheet and a consent form were sent to providers of all participating centres. The research team called and/or visited all centres to introduce the study aims, objectives and participation requirements. A focal representative from each centre was identified and charged with coordinating data collection logistics in the respective centre. That representative was mainly front-desk personnel or a secretary that had no care contact with patients (and thus are ineligible for participation in the survey) and is not part of the management or administration team. Consenting centres received a survey package that included surveys corresponding to the number of health-care providers at the centre. Participant providers were instructed to complete the questionnaire at a place where they can have privacy and to place them once completed in an enclosed envelope without including any identifiers. A courier company collected sealed envelopes from participating centres and returned them to the research team. Two weeks after initial receipt, the centres received a follow-up reminder phone call with a third and final phone reminder done after 6 weeks.

### Survey instrument

The questionnaire included two sections: the *first* assessing the readiness of clinical providers to use quality and safety indicators and the *second* section assessing the safety attitude at the primary care centre. For the assessment of providers’ readiness for and acceptability of using performance reporting and quality enhancement activities, the Readiness for Organization Change (ROC) scale by Holt et al. was adopted [14]. The 26-item scale was used with the consent of the authors with some slight modification by the research team in order to fit the context of primary health care in Lebanon. Change in this study was defined as the adoption of primary health-care performance indicators. The questionnaire includes four subscales measuring the following:

- Appropriateness of performance reporting (10 questions): assesses employees’ perception of the extent of appropriateness, need and legitimacy of the proposed change.
- Management support (six questions): measures employees’ perception of the degree to which their managers and leaders are committed to support them through the implementation of the upcoming change.
- Efficacy (six questions): measures the degree to which employees’ feel that they possess the skills to carry out required tasks and activities related to the change.
- Personal valence (four questions): refers to employees’ perception of the degree they will potentially benefit (or not) prospective change should it be implemented.

Factor analysis was conducted to evaluate the scale reliability in the context of PHCCs in Lebanon. Based on the scale reliability and internal consistency measures, one item (‘when this change is implemented, I don’t believe there is anything for me to gain’) was reallocated from the ‘appropriateness’ construct to the ‘personal valence’ construct. This item fits better with personal valence since it directly assess the extent to which the providers feel they will benefit from the prospective change. Furthermore, the results of two items (‘it doesn’t make sense for us to initiate this change’ and ‘the time we are spending on this change should be spent on something else’) were dropped from the ‘appropriateness’ construct in addition to one item (‘after this change, I expect to be recognized more for the work I do’) from the ‘personal valence’ construct. The specified items are excluded from subsequent analyses because they did not load with the other items in the scale due to context-specific environment. For the assessment of the safety attitude in the centres, a series of questions relating to safety attitude and adapted from the Agency of Health Research and Quality (AHRQ) Safety Attitude Questionnaire (SAQ)-Ambulatory version were utilized. The translation of the scale items followed Kaya’s methodology of translating the SAQ instrument to the Turkish language [25]. The original ROC and SAQ scales were translated into Arabic by the first author (MA). Then, this Arabic version was translated back into English by an independent translator who had never seen the original version before. The original scales and back-translation were compared by two experts in the field. Since the experts concluded that all items in them have the same meaning, the Arabic translation was accepted as valid. The survey was administered in either English or Arabic languages based on the preference of the provider.

### Data analysis, reliability and validity

For the ROC scale, the mean score of items for each subscale measure was calculated after reverse scoring negatively worded items. For the SAQ scale, responses were first converted to a 100-point scale as follows: Strongly Disagree = 0, Disagree = 25, Neutral = 50, Agree = 75, Strongly Agree = 100. Negatively worded items were reverse-scored, and responses to each item were summed up then divided by the number of items in that scale to create a scale score that ranged from 0 to 100. Scores are reported as the percentage of respondents who have positive attitudes towards each factor (score more than or equal to 75, i.e. agree or strongly agree). Scale reliability and internal consistency were confirmed using Cronbach’s alpha (α). Different items measuring the same concept were supposed to yield an internal reliability greater than 0.6 [26]. Confirmatory factor analysis (CFA) was used to test the psychometric soundness of the ROC scale across the four domain structures.

Univariate analysis, frequency percent mean and standard deviations were then carried out to describe the primary health-care providers’ and centres’ characteristics, as well as responses of centres’ providers to the survey. The second level of analysis was aimed at investigating the association between the readiness to implement quality indicators and safety attitude on the one hand and providers’ and centres’ characteristics on the other. Student *t*-tests and ANOVA *f* tests with Bonferroni correction were used to assess the statistical significance of the associations depending on the nature of the variables. Lastly, different logistic regression models with stepwise selection were constructed with each of the four readiness subscales entered as dependent variables after transformation into categorical variables (lowest through mean score categorized as not ready and any score > mean categorized as ready). The independent variables included organizational and individual characteristics. All analyses were carried at the 0.05 significance level and SPSS v20 was used.

## Results

Out of the 123 centres invited to participate in this study, 108 (88 %) agreed to participate. The 15 centres that did not agree belonged to one network whose administration decided that resources are stretched and participation could not be supported at the time of the study. Of the 108 centres agreeing to participate and receiving the survey questionnaires, 92 (85 %) responded. Non-respondent centres either were late in returning the questionnaires or they ceased their operation during the study period. In terms of providers, the research team received 1 012 completed surveys (47 % of the total population). Sixty-nine surveys were excluded because they either did not match the inclusion criteria, or 50 % or more of the questions were not completed. The final dataset included data from 943 questionnaires with a corresponding response rate of 44 %, comparable to other studies conducted in similar settings [27–29]. The highest response rate was from nurses (82 %) followed by specialists (43 %). Dentists, general practitioners and allied health professionals had comparable response rates of 36 %, 35 % and 34 %, respectively.

### Participants and construct validity of ROC scale

As observed in Table 1, a third of participating primary health-care providers were specialists (33 %), followed by nurses (25 %), allied health professionals (18 %) and dentists and family physicians (12 % each). The respondents were equally distributed across gender with 60 % of respondents falling in the above 40 age range. Three quarters of respondents were married (74 %) and working on full-time (38 %) or part-time bases (43 %). Three quarters of respondents (76 %) had over 6 years of experience in the primary health-care setting, and more than half (54 %) worked in health centres for non-governmental organizations (NGOs). From a self-rated perspective, a little more than half (55 %) of participating health-care providers at the sampled centres indicated that they serve a population of medium socioeconomic status.

**Table 1:** Characteristics of primary health-care centres and providers (*N* = 943)

Variable	Frequency (n)	Percentage (%)^a^
Individual characteristics		
Gender		
Male	443	49.3
Female	456	50.7
Age		
<30 years	100	11.1
30–39 years	262	29.0
40–49 years	292	32.4
>50 years	248	27.5
Marital status		
Married	646	73.9
Single	204	23.3
Other	24	2.7
Position in centre		
Family physicians	117	12.4
Specialists	311	33.0
Dentists	110	11.7
Nurses	236	25.0
Allied health professionals	169	17.9
Experience in primary care		
Less than 1 year	21	2.2
1–5 years	199	21.2
6–10 years	214	22.8
11–15 years	169	18.0
More than 15 years	334	35.6
Experience in current position		
Less than 1 year	90	9.7
1–5 years	385	41.5
6–10 years	211	22.8
11–15 years	118	12.7
More than 15 years	123	13.3
Employment status^b^		
Full-time	354	38.0
Part-time	396	42.5
Temporary/casual	181	19.4
Centre characteristics		
Governorate		
Beirut	136	14.4
Mount Lebanon	229	24.3
North	193	20.5
Bekaa	138	14.6
South	177	18.8
Nabatieh	70	7.4
Ownership type		
Public	206	21.8
NGO	506	53.7
Religious	231	24.5
Socioeconomic status of target population		
Good	9	1.1
Medium	461	54.8
Medium to low	114	13.6
Low	257	30.6

^a^Missing data were not included in the calculation of the reported percentages

^b^Full-time employment defined as working for 35 h or more per week; part-time employment defined as working for a fixed number of hours but less than 35 h per week; temporary/casual employment defined as not working for a fixed employment period and no fixed number of hours per week

A confirmatory factor analysis tested the validity of the four-factor structure of the ROC scale. Internal consistency was good for the four scales: appropriateness (alpha = 0.85), management support (alpha = 0.81), efficacy (alpha = 0.70) and personal valence (alpha = 0.66) (Table 2). The Appendix shows the items in each scale.

**Table 2:** Distribution of PHC respondents by ROC subscale scores and type

ROC subscale score (α)/type of provider	Generalists (*n* = 117)	Specialists (*n* = 311)	Dentists (*n* = 110)	Nurses (*n* = 236)	Allied health prof. (*n* = 169)	Total sample (*n* = 943)
Appropriateness^a^ (α = 0.85)						
% Ready	65.5	72.5	75.2	77.4	83.4	75.2
Mean score (SD)	5.14 (0.89)	5.20 (0.79)	5.29 (0.86)	5.32 (0.66)	5.38 (0.60)	5.26 (0.75)
Efficacy (α = 0.70)						
% Ready	64.3	63.9	70.6	66.2	75.0	67.3
Mean score (SD)	5.13 (0.75)	5.06 (0.81)	5.17 (0.88)	5.08 (0.70)	5.25 (0.69)	5.12 (0.76)
Management^b,c^ (α = 0.81)						
% Ready	58.8	57.3	62.4	63.5	71.4	62.2
Mean score (SD)	4.97 (0.94)	4.88 (0.95)	5.08 (0.90)	5.09 (0.82)	5.21 (0.84)	5.02 (0.90)
Personal valence^c,d^ (α = 0.66)						
% Ready	71.1	65.1	67.3	60.1	78.9	67.3
Mean score (SD)	5.18 (1.03)	5.04 (1.17)	5.17 (1.00)	5.08 (0.98)	5.40 (0.76)	5.14 (1.02)
Overall						
% Ready						66.3
Mean score (SD)						5.15 (0.66)

Readiness attitudes were defined as having scale scores >5/6, the equivalent of agree or strongly agree on the Likert scale used for the response options

Statistical comparisons are based on the mean scores

^a^ *P* < 0.05 based on ANOVA. Bonferroni’s multiple range test indicated that the mean scores for generalists and allied health professionals differed significantly

^b^ *P* < 0.001 based on ANOVA. Bonferroni’s multiple range test indicated that the mean scores for specialists and nurses differed significantly

^c^ *P* < 0.001 based on ANOVA. Bonferroni’s multiple range test indicated that the mean scores for specialists and allied health professionals differed significantly

^d^ *P* < 0.001 based on ANOVA. Bonferroni’s multiple range test indicated that the mean scores for nurses and allied health professionals differed significantly

### Centre and individual characteristics and readiness for organizational change

On average, two thirds (66 %) of the providers indicated that they are ready for the implementation of quality and patient safety indicators in their respective centres (Table 2). The highest readiness subscale was related to the appropriateness of performance measurement as a change in the centre (75 %) followed by personal valence and efficacy (both at 67 %). The lowest readiness score was reported on the ‘management support’ subscale with 62.2 % of respondents indicating supportive management that enhances their readiness for reporting quality and safety indicators.

In comparing the readiness of providers to implement the quality indicators, 66 % of generalists showed a positive perception for appropriateness of the introduction performance measurements, compared to 83 % of allied health professionals (Table 2). There were statistically significant differences in mean scores between the two aforementioned categories of providers (5.14 ± 0.89 versus 5.38 ± 0.60, respectively, *P* < 0.05). The percentage of specialists (57 %) with positive management support score was lower than that of allied health professionals (71 %) and nurses (63.5 %). The difference in the mean score on this EOC subscale was significant (4.88 ± 0.95 versus 5.21 ± 0.84 and 5.09 ± 0.82, respectively, *P* < 0.001). Furthermore, analysis reveals a significant difference in the mean scores of the personal valence subscale of ROC between allied health professionals and nurses (5.40 ± 0.76 versus 5.08 ± 0.98, respectively, *P* < 0.001). Overall, the comparison between the various types of providers reveals that the physicians, whether generalists or specialists, were relatively less ready than other care providers on all ROC subscales, except for personal valence which was lowest among nurses. Allied health professionals appear to be the readiest for the implementation of quality and patient safety indicators compared to their colleagues belonging to other professional groups.

In regard to the SAQ, respondents displayed a high percentage of agreement on the various SAQ items (Table 3), most notably the following: reinforcement of patient safety at the centre (94.1 %), encouragement to report patient safety concerns (89.6 %) and feeling safe to be treated at the centre (86.6 %). In contrast, lowest agreement proportions were noted for the following: not punishing staff for reported errors and incident reports (12.8 %), belief in the negative consequences of lack in continuity of care process (41.1 %) and perception of adequacy in medical equipment (48.1 %). Note that the overall average agreement on all items of the SAQ was 83.5 % with a Cronbach alpha of 0.8.

**Table 3:** Safety attitude scale descriptives

SAQ scale item	*N*	Mean	SD	% Positive^a^	Skewness	Kurtosis
Patient safety is constantly reinforced as the priority in this centre	931	4.50	0.70	94.1	−1.858	5.475
Providers in this centre are encouraged to report any patient safety concerns they may have	911	4.30	0.77	89.6	−1.432	3.435
I would feel safe being treated here as a patient	931	4.25	0.80	86.6	−1.254	2.353
All the personnel in this office take responsibility for patient safety	916	4.14	0.89	84.2	−1.285	2.005
The proper channels to direct questions regarding patient safety in this office are made clear in this centre	920	4.10	0.82	84.0	−1.116	1.947
The culture in this office makes it easy to learn from errors of others	924	4.05	0.84	82.0	−1.102	1.818
Changes are necessary to encourage providers to discuss errors	916	4.10	0.84	81.7	−1.031	1.582
Medical errors are handled appropriately in this centre	915	4.08	0.88	81.3	−1.166	1.882
I receive appropriate feedback about my performance	916	4.05	0.90	80.8	−1.083	1.224
There is widespread adherence to clinical guidelines and evidence-based criteria regarding patient safety in this centre	899	4.06	0.83	80.5	−1.018	1.721
Briefing other personnel before a procedure (e.g. biopsy) is important for patient safety	881	3.92	0.92	72.4	−.814	.663
All the necessary information for diagnostic and therapeutic decisions is routinely available to providers in this centre	925	3.77	0.95	68.9	−.651	−.051
Briefings are common in this centre	871	3.62	0.94	60.0	−.623	.365
The medical equipment in this centre is adequate	927	3.23	1.18	48.1	−.193	−1.015
Disruptions in the continuity of care (e.g. shift changes, and patient transfers) can be detrimental to patient safety	896	2.96	1.26	41.1	−.051	−1.153
Providers are not punished for errors reported through incident reports	909	2.28	1.14	12.8	.700	−.140
^b^There exists some barriers that compromise the safety of patients in this centre	913	2.01	1.05	11.6	.990	.305
^b^Providers frequently disregard rules or guidelines (e.g. hand washing, treatment protocols/clinical pathways and sterile field) that are established for this centre	924	1.91	1.05	9.4	1.103	.559
^b^Errors that had the potential to harm patients were made in this centre	917	1.97	1.00	8.6	.924	.291
Safety attitude climate total score	938	3.87	0.43	83.5	*α* = 0.80	

^a^Percent positive scores are the percentage of respondents who have positive attitudes towards each factor: score more than or equal to 75 for item scores and more than or equal to 62.5 for total score

^b^Reverse-scored item

Table 4 displays a bivariate analysis of the ROC of providers to adopt performance measures across a number of variables. The table reveals that ROC was positively associated with the safety attitude at a significant level (*P* < 0.001) across all domains of readiness. Furthermore, providers with more than 15 years of experience reported a significantly higher mean score on the efficacy subscale compared to those with less than 15 years of experience. Similarly, full-time employment status was significantly associated with all domains of readiness except for personal valence. On average, permanent full-time providers had higher mean scores compared to temporary or casual workers. Regarding centre ownership, providers at centres belonging to religious institutions had higher mean scores on personal valence compared to publicly owned centres and NGO-owned centres.

**Table 4:** Readiness of implementing quality indicators bivariate analysis

Variable	Mean (SD)							
	Appropriateness^a,b^		Efficacy^c,d^		Management^e^		Personal valence^f,g^	
Safety attitude								
Negative attitude	5.09	(0.79)*	4.93	(0.81)*	4.73	(0.93)*	5.02	(1.04)*
Positive attitude	5.53	(0.59)	5.41	(0.59)	5.47	(0.63)	5.35	(0.95)
Gender								
Male	5.19	(0.82)*	5.08	(0.80)	4.97	(0.93)	5.14	(1.04)
Female	5.36	(0.66)	5.19	(0.72)	5.09	(0.86)	5.20	(0.97)
Age								
<30 years	5.26	(0.80)	5.07	(0.82)	4.93	(0.89)	5.01	(1.09)
30–39 years	5.28	(0.73)	5.15	(0.69)	5.08	(0.87)	5.20	(0.94)
40–49 years	5.32	(0.69)	5.18	(0.78)	5.08	(0.90)	5.26	(0.92)
>50 years	5.20	(0.81)	5.09	(0.73)	4.98	(0.92)	5.19	(0.98)
Governorate								
Beirut	5.19	(0.76)**	5.09	(0.73)**	4.98	(0.84)*	5.18	(0.81)*
Mount Lebanon	5.24	(0.72)	5.20	(0.77)	5.06	(0.84)	5.35	(0.77)
North	5.41	(0.69)	5.23	(0.78)	5.16	(0.85)	5.23	(1.09)
Bekaa	5.13	(0.86)	4.96	(0.74)	4.82	(0.97)	4.78	(1.32)
South	5.32	(0.71)	5.10	(0.67)	5.15	(0.82)	5.00	(1.12)
Nabatieh	5.20	(0.86)	5.01	(0.95)	4.70	(1.21)	5.25	(0.83)
Marital status								
Married	5.28	(0.75)	5.16	(0.73)	5.05	(0.90)	5.19	(0.99)
Single	5.25	(0.73)	5.10	(0.77)	5.07	(0.85)	5.24	(0.91)
Other	5.48	(0.54)	5.23	(0.68)	5.01	(0.85)	5.10	(0.88)
Experience in domain								
Less the 1 year	4.97	(1.23)	4.96	(0.98)	4.71	(1.02)	4.90	(1.33)
1–5 years	5.26	(0.76)	5.07	(0.76)	4.98	(0.89)	5.03	(1.08)
6–10 years	5.19	(0.77)	5.12	(0.79)	4.99	(0.95)	5.17	(1.09)
11–15 years	5.30	(0.70)	5.15	(0.77)	5.04	(0.85)	5.15	(1.04)
More than 15 years	5.32	(0.73)	5.15	(0.73)	5.09	(0.89)	5.23	(0.91)
Experience in centre								
Less the 1 year	5.15	(0.95)	5.01	(0.93)*	5.04	(0.94)	5.05	(1.05)
1–5 years	5.27	(0.74)	5.16	(0.75)	5.00	(0.90)	5.15	(0.95)
6–10 years	5.30	(0.72)	5.24	(0.64)	5.11	(0.86)	5.28	(1.02)
11–15 years	5.33	(0.70)	5.18	(0.69)	5.02	(0.98)	5.17	(0.96)
More than 15 years	5.28	(0.64)	4.89	(0.82)	5.00	(0.84)	5.07	(1.18)
Employment status								
Full-time	5.37	(0.69)*	5.19	(0.71)**	5.14	(0.87)*	5.17	(0.99)
Part-time	5.20	(0.77)	5.12	(0.75)	5.01	(0.83)	5.19	(1.03)
Temporary/casual	5.19	(0.83)	5.02	(0.82)	4.83	(1.02)	5.12	(0.95)
Centre ownership								
Public	5.28	(0.76)	5.20	(0.78)	5.09	(0.86)	4.94	(1.26)**
NG0	5.28	(0.75)	5.12	(0.77)	5.00	(0.91)	5.19	(0.96)
Religious	5.23	(0.74)	5.05	(0.73)	5.03	(0.90)	5.24	(0.90)

*Significance level at *P* < 0.001; **significance level at *P* < 0.05

^a^ *P* < 0.001 based on ANOVA. Bonferroni’s multiple range test indicated that the mean scores for full-time and casual workers differed significantly

^b^ *P* < 0.001 based on ANOVA. Bonferroni’s multiple range test indicated that the mean scores for part-time and casual workers differed significantly

^c^ *P* < 0.05 based on ANOVA. Bonferroni’s multiple range test indicated that the mean scores for full-time and casual workers differed significantly

^d^ *P* < 0.001 based on ANOVA. Bonferroni’s multiple range test indicated that the mean scores for those with experience of more than 15 years and each of the other experiences differed significantly

^e^ *P* < 0.001 based on ANOVA. Bonferroni’s multiple range test indicated that the mean scores for full-time and casual workers differed significantly

^f^ *P* < 0.05 based on ANOVA. Bonferroni’s multiple range test indicated that the mean scores for public and religious differed significantly

^g^ *P* < 0.05 based on ANOVA. Bonferroni’s multiple range test indicated that the mean scores for public and NGO differed significantly

### Multinomial logistic regression

A multinomial logistic regression analysis was carried to further examine the elements significantly associated with providers’ readiness to implement performance measures at their respective centres (Table 5). The safety attitude at the centre remained positively associated with all four subscales of readiness for change. The highest odds was noted at the management subscale: providers with a positive safety attitude had 7.1 times the odds of reporting a supportive management for the proposed change as compared to those with negative safety attitude (95 % CI: 4.3–11.7; *P* value <0.001). Likewise, providers employed on a full-time basis had 1.7 (95 % CI: 1.1–2.7; *P* value 0.014) and 2.2 (95 % CI: 1.3–3.7; *P* value 0.002) times the odds of reporting self-perceived skills to execute the tasks and activities associated with the proposed change when compared to part-timers and temporary/casual workers, respectively. In addition, full-time workers had 2.2 (95 % CI: 1.4–3.4; *P* value 0.001) and 1.7 (95 % CI: 1.0–2.9; *P* value 0.036) times the odds of understanding the appropriateness of the performance measures as compared to part-timers and temporary/casual workers, respectively. Regarding the occupation of the providers, family physicians, specialists, dentists and allied health professionals had higher odds (2.7, 2.2, 2.7 and 2.0, respectively) of reporting higher levels of efficacy when compared to nurses. With respect to appropriateness of the change, dentists had 2.4 (95 % CI: 1.3–4.4; *P* value 0.007) times the odds of noting the change as appropriate compared to nurses. As for the personal valence, allied health professionals had 1.6 (95 % CI: 1.0–2.5; *P* value 0.038) times the odds of feeling that they will benefit from the proposed change when compared to nurses.

**Table 5:** Regression model testing for predictors of readiness for change

Variable	Readiness for change											
	Appropriateness			Efficacy			Management			Personal valence		
	OR	CI	*P* value	OR	CI	*P* value	OR	CI	*P* value	OR	CI	*P* value
Safety attitude												
Positive attitude	2.708	(1.796–4.083)	<0.001	1.755	(1.189–2.593)	0.005	7.050	(4.264–11.656)	<0.001	2.427	(1.618–3.641)	<0.001
Negative attitude	1			1			1			1		
Gender												
Female	1.346	(0.980–1.848)	0.066	1.249	(0.912–1.710)	0.166	1.103	(0.797–1.526)	0.555	1.164	(0.848–1.599)	0.347
Male	1			1			1			1		
Region												
Non-major city (<200 k inhabitants)	0.799	(0.596–1.070)	0.132	0.832	(0.623–1.112)	0.215	0.785	(0.581–1.060)	0.115	1.150	(0.858–1.541)	0.351
Major city (>200 k inhabitants)	1			1			1			1		
Experience in domain												
Less the 1 year	1.017	(0.382–2.694)	0.974	0.946	(0.358–2.498)	0.910	0.699	(0.248–1.790)	0.499	1.294	(0.479–3.498)	0.611
1–5 years	0.817	(0.556–1.201)	0.305	0.919	(0.627–1.347)	0.666	0.864	(0.581–1.286)	0.472	0.728	(0.494–1.074)	0.109
6–10 years	0.639	(0.440–0.928)	0.019	0.953	(0.661–1.374)	0.796	0.812	(0.555–1.187)	0.282	1.225	(0.849–1.768)	0.277
11–15 years	0.904	(0.608–1.345)	0.619	0.986	(0.664–1.463)	0.944	0.791	(0.526–1.189)	0.260	0.929	(0.623–1.383)	0.716
More than 15 years	1			1			1			1		
Employment status												
Part-time	0.457	(0.294–0.712)	0.001	1.495	(0.999–2.237)	0.052	0.660	(0.424–1.028)	0.066	0.913	(0.592–1.408)	0.680
Temporary/casual	0.574	(0.342–0.964)	0.036	0.999	(0.714–0.398)	0.997	0.673	(0.398–1.139)	0.140	0.716	(0.429–1.195)	0.201
Full-time	1			1			1			1		
Centre ownership												
Public	1.232	(0.820–1.852)	0.314	0.576	(0.371–0.896)	0.014	1.093	(0.720–1.658)	0.676	0.973	(0.650–1.458)	0.895
NGO	0.905	(0.645–1.270)	0.562	0.448	(0.267–0.752)	0.002	1.046	(0.739–1.480)	0.801	0.943	(0.673–1.322)	0.735
Religious	1			1			1			1		
Providers												
Family physician	1.490	(0.819–2.713)	0.192	2.702	(1.483–4.921)	0.001	1.198	(0.657–2.187)	0.556	1.464	(0.811–2.645)	0.206
Specialists	1.495	(0.875–2.554)	0.141	2.165	(1.265–3.705)	0.005	1.103	(0.642–1.893)	0.723	1.530	(0.902–2.595)	0.114
Dentist	2.366	(1.272–4.402)	0.007	2.700	(1.456–5.008)	0.002	1.547	(0.828–2.891)	0.171	1.577	(0.856–2.906)	0.144
Allied health professionals	1.097	(0.708–1.701)	0.678	1.954	(1.260–3.030)	0.003	1.540	(0.979–2.423)	0.062	1.587	(1.026–2.455)	0.038
Nurses	1			1			1			1		

## Discussion

Organizational change cannot be effectively realized without the appropriate consideration of the willingness and readiness of the human resources that have the capacity to either promote or hinder any change [9]. Having that in mind, the introduction of performance improvement measures does not only require an assessment of the readiness of providers for the proposed change but also sensitivity to the needs of the various professional groups across the various dimensions of change for it to be successful. From that end, the purpose of this study is to inform PHCC managers and policy-makers, as well as researchers in health systems, of the readiness of clinical providers for the introduction of performance improvement and patient safety indicators in PHC centres.

The overall readiness for change scale indicated that two thirds of the clinical providers participating in the survey showed readiness for the implementation of performance indicators in their PHC centres. Further dissection of the results across the four assessed domains of readiness reveals that three out of four PHC providers believe that the introduction of performance measures in the health centres is legitimate and appropriate for the organization to meet its objectives. In the setting of primary health care in Lebanon, the recorded high level of readiness among clinical providers can be explained by the initiative launched by the MoPH in Lebanon a few years back that aims at raising the standards of primary health care in Lebanon and the introduction of a national accreditation system for PHC centres similar to that of hospitals. On that front, the ministry launched a series of workshops to promote an environment of continuous quality improvement among clinical providers working at the PHC sector in Lebanon. In addition, a selected group of centres underwent a first cycle of accreditation with upcoming cycles of accreditation for more centres [24]. Thus, our finding reflects on the success of the MoPH capacity-building campaign in preparing managers and providers to endorse quality initiatives. Nonetheless, the level of readiness among clinical providers for organizational change varies across domains of change and professional groups [12, 15].

Perhaps what is most worrisome are the readiness scales of the two most important providers in the PHC systems, physicians and nurses. The former group reveals relatively lower readiness scores across all scales except for personal valence while nurses were found to be least ready for change among all providers in the regression analysis. Such findings raise serious questions on the true commitment of the largest providers of care to the proposed changes by the MoPH. Some will argue that many physicians (generalist and specialists) may dedicate part of their time at the PHC centre and thus would be less committed and acquainted with the proposed changes. Such a finding is confirmed in the regression analysis revealing lower odds of readiness for part-time and casual providers at PHCs, many of which are physicians. Yet, genuine concerns should be expressed on the extent of readiness for nurses to endorse performance quality and patient safety indicators. Nurses as a group often complain that they are overworked and feel that the onus of administrative work related to reporting patient safety and quality indicators would fall on their shoulders [30–32]. Policy- and decision-makers are advised to pay particular attention to enhancing the readiness of nurses for change through targeted training sessions, workshops and careful restructuring of their work duties in order to provide them the time necessary to carry out administrative requirement related to reporting quality and safety indicators.

Clinical providers’ attitude about topics relevant to the safety climate in the PHC setting revealed a moderate to high score with over 83 % of providers having a positive safety attitude. Our findings compare favourably with similar ambulatory settings where the SAQ-A scale was administered to outpatient providers [33]; our scores were generally higher. Similarly, the safety climate among primary care providers was higher as compared to peers in Turkish hospitals [25]. This could be linked to the positive safety atmosphere created in many of the PHC centres in preparation for the upcoming accreditation surveys especially that such an association was seen in similar settings in literature [34–37]. Nevertheless, there remain some deficiencies in employees’ perception of safety climate at their centres, and the onus is on their leaders and managers to work on changing these perceptions. Most importantly, surveyed providers report the presence of a punitive and blaming culture in case of error or near-miss reporting at PHC centres. Such a finding is quite worrisome for two reasons: first, it presents a major obstacle for improving quality and safety of care at PHC centres and reflects a culture of fear in which errors are hidden or under-reported. Second, our findings reflect that any improvement in employees’ scores on the SAQ are directly correlated to their readiness across the various readiness subscales both in health care and other settings [16, 38, 39]. Creating a just and non-punitive culture would require a number of changes including the following: strengthening team work, fostering shared accountability, bolstering understanding and management abilities of behavioural choices, enhancing system thinking and safe system design and creating a proactive learning culture aiming at detecting latent errors. The onus is on the Lebanese MoPH and the managers and directors of centres to transform their work culture from a punitive and blaming culture to that of justice and shared responsibility in which errors are attributed to deficiencies in care systems rather than in individuals.

Furthermore, providers’ diverse characteristics explained the variance in readiness for organizational change. Being in part-time or casual employment was associated with lower levels of readiness. This association highly correlates with evidence from literature [40] attributed to the fact that part-timers tend to be less involved and committed to the practice [41]. In addition, allied health professionals were generally more ready for change when compared to physicians, particularly in terms of appropriateness, management support and personal valence of change thought. This could be explained by the expanded involvement of allied health professionals in quality improvement initiatives and their attendance of educational activities organized by the MoPH, as compared to physicians who generally are too busy to participate in such activities. However, other characteristics were not statistically associated with the level of providers’ readiness to implement quality indicators. The results did not strongly reinforce the relationship between the number of years that the providers worked in the primary care domain and the level of individual readiness. This correlates with findings from literature indicating the absence of such a relationship [40]. Further, the results showed that the PHC centre ownership was not related to readiness for change. There was one exception: providers working at government-owned facilities showed variance in some domains of readiness when compared to peers in privately owned (NGO and religious) institutions. Such findings are not surprising in the scope that public and not-for-profit care organizations provide the same level of care quality despite the minor different goals and strategies adopted by the two sectors [42] which could explain the slight variation across some domains of readiness.

There are some limitations that need to be acknowledged in this study. First, 92 out of 123 PHC centres participated in the study. Although preliminary assessment showed that these centres tend to have comparable characteristics to that of participating centres, it cannot be ascertained that the health-care providers in non-participating centres have the same attitude towards accreditation and quality indicators as that of the responding centres. Also, the self-reported nature of the survey may contribute to some sort of bias related to social desirability associated with the general appreciation of the importance of quality indicators. Nevertheless, frank responses to specific items of the scale (such as the non-punitive culture) may attenuate such concerns. More specific research into the acceptability and satisfaction of the providers after the introduction of the performance indicators in this specific context might be valuable to capture the perspectives of all primary health-care providers that are expected to vary according to roles and responsibilities.

In conclusion, health-care providers in PHC centres in Lebanon are ready to engage in employing quality and patient safety indicators. This is a key finding given the active efforts by the MoPH to strengthen the quality culture in the PHC sector through various strategies.

## Appendix

Readiness for change factors (Total = 26 items) (Holt et al. [14]):

### Appropriateness

*Measures the extent to which one feels that the change effort was legitimate and appropriate for the organization to meet its objectives.*

1. It doesn’t make much sense for us to initiate this change. (R) (dropped)
2. I think that the organization will benefit from this change.
3. This change makes my job easier.
4. This change will improve our organization’s overall efficiency.
5. There are legitimate reasons for us to make this change.
6. When this change is implemented, I don’t believe there is anything for me to gain. (R) (moved to ‘personal valence’ construct)
7. There are a number of rational reasons for this change to be made.
8. In the long run, I feel it will be worthwhile for me if the organization adopts this change.
9. The time we are spending on this change should be spent on something else. (R) (dropped)
10. This change matches the priorities of our organization.

### Management support

*Measures the extent to which one feels that the organization’s leadership and management are committed to and support implementation of the prospective change.*

1. Our senior leaders have encouraged all of us to embrace this change.
2. Our organization’s top decision-makers have put all their support behind this change effort.
3. Every senior manager has stressed the importance of this change.
4. I think we are spending a lot of time on this change when the senior managers don’t even want it implemented. (R)
5. This organization’s most senior leader is committed to this change.
6. Management has sent a clear signal this organization is going to change.

### Efficacy

*Measures the extent to which one feels that he or she has the skills and is able to execute the tasks and activities that are associated with the implementation of the prospective change.*

1. I do not anticipate any problems adjusting to the work I will have when this change is adopted.
2. When we implement this change, I feel I can handle it with ease.
3. When I set my mind to it, I can learn everything that will be required when this change is adopted.
4. There are some tasks that will be required when we change I don’t think I can do well. (R)
5. I have the skills that are needed to make this change work.
6. My past experiences make me confident that I will be able to perform successfully after this change is made.

### Personal valence

*Measures the extent to which one feels that he or she will benefit from the implementation of the prospective change.*

1. I am worried I will lose some of my status in the organization when this change is implemented. (R)
2. This change will disrupt many of the personal relationships I have developed. (R)
3. My future in this job will be limited because of this change. (R)
4. After this change, I expect to be recognized more for the work I do. (dropped)

## References

[1] WHO. Declaration of the Alma-Ata: international conference on primary health care. 1978.

[2] WHO. The World Health Report 2008 - primary health care (now more than ever). 2008.

[3] Kohn LT, Corrigan JM, Donaldson MS (2002). To err is human: building a safer health system. J Interprof Care.

[4] Saleh SS, Alameddine MS, Natafgi NM (2014). Beyond accreditation: a multi-track quality-enhancing strategy for primary health care in low- and middle-income countries. Int J Health Serv.

[5] Gibberd R, Hancock S, Howley P, Richards K (2004). Using indicators to quantify the potential to improve the quality of health care. Int J Qual Health Care..

[6] Marshall MN, Shekelle PG, McGlynn EA, Campbell S, Brook RH, Roland MO (2003). Can health care quality indicators be transferred between countries?. Qual Saf Health Care.

[7] Campbell SM, Braspenning J, Hutchinson A, Marshall MN (2003). Research methods used in developing and applying quality indicators in primary care. BMJ.

[8] Holt DT, Helfrich CD, Hall CG, Weiner BJ (2010). Are you ready? How health professionals can comprehensively conceptualize readiness for change. J Gen Intern Med..

[9] Smith I (2005). Achieving readiness for organizational change. Libr Manag.

[10] Weiner BJ (2009). A theory of organizational readiness for change. Implement Sci.

[11] Eby LT, Adams DM, Russell JEA, Gaby SH (2000). Perceptions of organizational readiness for change: factors related to employees’ reactions to the implementation of team-based selling. Hum Relat.

[12] Cunningham CE, Woodward CA, Shannon HS, MacIntosh J, Lendrum B, Rosenbloom D (2002). Readiness for organizational change: a longitudinal study of workplace, psychological and behavioural correlates. J Occup Organ Psychol.

[13] Hamilton S, McLaren S, Mulhall A (2007). Assessing organisational readiness for change: use of diagnostic analysis prior to the implementation of a multidisciplinary assessment for acute stroke care. Implement Sci..

[14] Holt DT, Armenakis AA, Feild HS, Harris SG (2007). Readiness for organizational change: the systematic development of a scale. J Appl Behav Sci.

[15] Jennett P, Yeo M, Pauls M, Graham J (2003). Organizational readiness for telemedicine: implications for success and failure. J Telemed Telecare..

[16] Naveh E, Katz-Navon T, Stern Z (2006). Readiness to report medical treatment errors: the effects of safety procedures, safety information, and priority of safety. Med Care.

[17] Jones RA, Jimmieson NL, Griffiths A (2005). The impact of organizational culture and reshaping capabilities on change implementation success: the mediating role of readiness for change. J Manag Stud.

[18] Backer TE, David SL, Soucy G (1995). Reviewing the behavioral science knowledge base on technology transfer. Introduction. NIDA Res Monogr.

[19] Ammar W (2003). Health system and reform in Lebanon: Beirut.

[20] Ammar W (2009). Health beyond politics: first ed.

[21] Ministry of Public Health. Annual report on primary health care. 2012.

[22] Ministry of Public Health in Lebanon - Primary Health Care Directorate. Primary health care - first mid-year annual report. 2012.

[23] Accreditation Canada. Lebanon to invest in the quality of primary health care. 2009.

[24] Anonymous. The Ministry of Public Health finalizes its first round of primary health care accreditation. Al Moustaqbal Newspaper. 27 April 2011; Issue 3981, page 12.

[25] Kaya S, Barsbay S, Karabulut E (2010). The Turkish version of the safety attitudes questionnaire: psychometric properties and baseline data. Qual Saf Health Care.

[26] Field A (2000). Discovering statistics using SPSS for Windows.

[27] Wechsler H, Levine S, Idelson RK, Schor EL, Coakley E (1996). The physician’s role in health promotion revisited–a survey of primary care practitioners. N Engl J Med.

[28] Eriksson L, Nga NT, Malqvist M, Persson LA, Ewald U, Wallin L (2009). Evidence-based practice in neonatal health: knowledge among primary health care staff in northern Viet Nam. Hum Resour Health.

[29] Kushner RF (1995). Barriers to providing nutrition counseling by physicians: a survey of primary care practitioners. Prev Med.

[30] Storey C, Cheater F, Ford J, Leese B (2009). Retention of nurses in the primary and community care workforce after the age of 50 years: database analysis and literature review. J Adv Nurs.

[31] Storey C, Cheater F, Ford J, Leese B (2009). Retaining older nurses in primary care and the community. J Adv Nurs.

[32] Hayes LJ, O’Brien-Pallas L, Duffield C, Shamian J, Buchan J, Hughes F, Laschinger HK, North N (2012). Nurse turnover: a literature review - an update. Int J Nurs Stud.

[33] Modak I, Sexton JB, Lux TR, Helmreich RL, Thomas EJ (2007). Measuring safety culture in the ambulatory setting: the safety attitudes questionnaire–ambulatory version. J Gen Intern Med.

[34] Byrd HS, Barton FE, Orenstein HH, Rohrich RJ, Burns AJ, Hobar PC (2003). Safety and efficacy in an accredited outpatient plastic surgery facility: a review of 5316 consecutive cases. Plast Reconstr Surg.

[35] Rohrich RJ, White PF (2001). Safety of outpatient surgery: is mandatory accreditation of outpatient surgery centers enough?. Plast Reconstr Surg.

[36] Lapetina EM, Armstrong EM (2002). Preventing errors in the outpatient setting: a tale of three states. Health Aff (Millwood).

[37] Al Tehewy M, Salem B, Habil I, El Okda S (2009). Evaluation of accreditation program in non-governmental organizations’ health units in Egypt: short-term outcomes. Int J Qual Health Care.

[38] Hofmann DA, Stetzer A (1998). The role of safety climate and communication in accident interpretation: implications for learning from negative events. Acad Manage J.

[39] Zohar D (2000). A group-level model of safety climate: testing the effect of group climate on microaccidents in manufacturing jobs. J Appl Psychol.

[40] Christl B, Harris MF, Jayasinghe UW, Proudfoot J, Taggart J, Tan J (2010). Readiness for organisational change among general practice staff. Qual Saf Health Care.

[41] Madsen SR, Miller D, John CR (2005). Readiness for organizational change: do organizational commitment and social relationships in the workplace make a difference?. Hum Resour Dev Q.

[42] Amirkhanyan AA, Kim HJ, Lambright KT (2008). Does the public sector outperform the nonprofit and for-profit sectors? Evidence from a national panel study on nursing home quality and access. J Policy Anal Manage.

